# Effective playing time affects technical-tactical and physical parameters in football

**DOI:** 10.3389/fspor.2023.1229595

**Published:** 2023-08-08

**Authors:** Óscar Tojo, Konstantinos Spyrou, João Teixeira, Paulo Pereira, João Brito

**Affiliations:** ^1^ Portugal Football School, Federação Portuguesa de Futebol, Oeiras, Portugal; ^2^ UCAM Research Center for High Performance Sport, UCAM Universidad Católica de Murcia, Murcia, Spain; ^3^ Facultad de Deporte, UCAM Universidad Católica de Murcia, Murcia, Spain; ^4^ Departamento de Desporto e Saúde, Escola de Ciências e Tecnologia, Universidade de Évora, Évora, Portugal; ^5^ Comprehensive Health Research Centre (CHRC), Universidade de Évora, Évora, Portugal

**Keywords:** soccer, performance, external load, match-play, team-sports

## Abstract

Effective playing time has been debated as a topic of major concern in football. Thus, the current experimental study aimed to investigate the effects of effective playing time on technical-tactical and physical match parameters in football. One hundred and seventy-nine male highly trained football players (aged 27.9 ± 5.1 years) from twelve teams performed two different match-play conditions: 45 min of match-play without stopping the chronometer (T45), and 30 min of match-play by stopping the chronometer every time the ball was out-of-play (T30). T30 presented a significantly higher total time (49:30 vs. 45:00 min; *p *= <.001; ES = 0.76), effective playing time (28:70 vs. 26:80 min; *p *= <.001; ES = 0.62), and stopped time (20:60 vs. 18:20 min; *p *= 0.003; ES = 0.38) compared to T45. Total ball possession (54.4% vs. 45.6%; *p = *0.002) and 1/3 ball possession (55.3% vs. 44.7%; *p *= 0.018) was higher in T30 condition when compared to T45. Regarding match external load, total distance covered (4,899 vs. 4,481 m; *p *= <.001; ES = 0.71), moderate-speed running (607 vs. 557 m; *p *= 0.002; ES = 0.26) and high-speed running (202 vs. 170 m; *p *= <.001; ES = 0.33), high-speed activities (284 vs. 245 m; *p *= 0.003; ES = 0.24), accelerations (27 vs. 24; *p *= <.001; ES = 0.32), and decelerations (31 vs. 28; *p *= 0.005; ES = 0.26) were higher in T30 compared to T45. In conclusion, these findings suggest that higher effective playing time may affect technical-tactical and physical parameters during football games.

## 1. Introduction

Football match-play has been considered as a complex, self-organised, unstable, unpredictable, and highly dynamic system, in which coaches and players try to keep the stability of their own attacking and defending system and to disorganize the opponents' structure (1–3). However, several contextual factors, such as match location, competition features, team ranking, level of play, match outcome, and style of play may influence individual and team behaviors (4–8). Consequently, players must adapt physically, physiologically, and psychologically to different match-related contextual factors, which may lead to changes and variations in technical-tactical actions and patterns during match-play (3).

Football interruptions are a main characteristic of a match and may underline the intermittent nature of sport (9–11). Recently, effective playing time has been debated as a topic of major concern by several stakeholders in football (10–13), as official matches last for >90 min, however several interruptions, such as substitutions, fouls, injuries, goals, or the ball being out-of-play could negatively affect the effective playing time (9, 14). For example, it has been found in the Portuguese League that effective playing time was on average −49 min of total playing time (i.e., 90 min), which is consistently lower compared to other European Leagues (i.e., Germany, Spain, France, England, and Italy) and the Champions League (15). Given that, a recent study (16) considering the effective playing time, distinguishing the two main phases of the game (i.e., possession or non-possession of the ball), and normalizing the total distance to meters per minute (m·min^−1^), found that teams run more without possession than in the ball-possession, and the effective playing time and the amount of ball possession influenced the players’ running performance. Another study (17) investigated the effects of the Video Assistant Referee system (VAR) on the playing time, technical-tactical, and physical performance in soccer players, and found that the total playing time increased slightly when compared to three situations (without the intervention of VAR = 95.1 min, one intervention of VAR = 96.0 min, and 2–3 interventions of VAR = 99.1 min), however, effective playing time decreased significantly between without VAR and one intervention of the VAR (52.5 vs. 51.5 min). Considering technical-tactical parameters, in the same study (17) the total scored goals increased as the number of VAR intervention increased.

Regarding the match demands during effective playing time, professional players competed in the German Bundesliga covered on average 10% more total distance, and performed 13% more accelerations, while sprinted 7%–10% less in official matches with long (>65 min) compared to short (<50 min) effective playing time (12). Moreover, professional soccer players of the English Championship presented lower physical match demands comparing whole match metrics compared to the ball was in play metrics (18). Similar results found in youth elite soccer players (19). Considering effective playing time and contextual factors, such as team's level, match result and location, total distance was greater when playing at home, when the team was losing, and when the level of the opposing team was higher (20). However, more research is warranted about the effects of effective time on physical and technical-tactical parameters during football matches.

Other team sports (e.g., futsal, basketball) can be a benchmark for effective playing time, because the time is stopped when the ball is out-of-play. In football, however, several contextual variables may affect effective playing time. Therefore, the current study aimed to explore the differences on match-play time-related variables (i.e., total, effective, and stopped time), technical-tactical parameters (e.g., ball possession, shots to the goal), external load metrics, and other match-related events (i.e., medical assistance, goal-kicks, corners, throw-ins, kick-offs, free kicks) in two different match-play conditions: 45 min of match-play without stopping the chronometer when the ball was not in-play (T45), and 30 min of match play stopping the chronometer every time the ball was out-of-play (T30). The players, coaches, and referees were unaware about the way playing time had been counted, and only an external timekeeper was responsible for the time counting during match play. It was hypothesized that T30 would present higher time-related variables, technical-tactical parameters, and external load metrics when compared to T45.

## 2. Materials and methods

### 2.1. Study design

An experimental observational study was designed. Match-play data from 6 friendly games were collected. Matches were randomly divided into a 45-min half (T45; 45 min of match-play without stopping the chronometer when the ball was not in-play), or a 30-min effective-time half (T30; the chronometer was stopped every time the ball was out-of-play). An overall of six halves of T45 and six halves of T30 was analyzed for the current study. All games had official football referees (i.e., one main referee, and two assistant referees), but an external timekeeper was responsible for the time count during the match play. An official futsal timekeeper was responsible for the chronometer (Keenso PC3830A, USA), and provided no feedback to the participants during the experiment. Between each half, there was a 15-min half-time; during this break, no feedback has been provided to the participants by the timekeeper. All games were played by the end of the season 2021–2022.

### 2.2. Participants

One hundred and seventy-nine male amateur highly trained football players [i.e., Tier 3: Highly Trained/National Level (21)] (aged 27.9 ± 5.1 years) from twelve teams competing in the 4^th^ and 5^th^ tier in Portugal (regional level) participated in the experiment. All players had >18 years old and had been involved in 3–4 training sessions per week and one weekly official match during the season 2021–2022. Goalkeepers were excluded from the study because of the different technical-tactical and physical match demands. Prior to participation, the study design and objectives were carefully explained to the players, referees, and teams' staff. The participants then signed an informed consent. The study was approved by the Ethics Committee of the Portugal Football School (n°15/CEPFS/2022).

### 2.3. Procedures

All games were recorded using a 35-megapixel camera (Canon XA15Hd, Japan). The camera was placed above the field to record the entire pitch throughout the entire duration of the match. The matches were analysed and coded with LongoMatch software (Barcelona, Spain). Three categories of variables were used: time-related variables, match events related with stopped time, and offensive technical-tactical variables (Table 1).

**Table 1:** Time-related variables, match events related with stopped time and offensive technical-tactical variables analysed.

Variables	Definition
Time	
Total time	Total game time in each part
Effective time	Time of the ball is in play
Effective time (%)	The percentage of the time of the ball is in play divided by the total time
Stopped time	The time play is stopped
Match events	
Medical assistance	The time that is spent for medical assistance on-the-pitch
Goal-kick	The time that is spent for a goal kick
Corner	The time that is spent for a corner kick
Throw-in	The time that is spent for a thrown-in
Kick-off	The time that is spent for a kick-off
Free-kick	The time that is spent for a free kick
Substitutions	The time that is spent for substitutions
Technical-tactical	
Total possession	The actions of the team had in possession of the ball
1/3 possession	The actions of the team had possession of the ball in the last third of the offensive field^a^
Within area	The actions of the team had possession of the ball inside the penalty area
Shot within area	Shot on goal within the penalty area
Shot out of area	Shot on goal outside the penalty area

^a^ The last third of the field was defined as all the area of play from the edge of the penalty area's semicircular until the final line.

The players wore GPS units (APEX, STATSports, Northern Ireland) with a sampling frequency of 10 Hz. Each player wore a tight vest with the GPS unit on the back of their upper body between the scapula as described by the manufacturer. The devices were activated 15 min prior to the start of each match; this period has been excluded from analyses, and only data from players playing full matches were included. Doppler derived speed data was exported from manufacturer software (STATSports Sonra 2.1.4) into intel(R) Core (TM) i7 for processing and to derive metrics. External load metrics included absolute total distance covered, moderate-speed running (14.4–19.8 km·h^−1^), high-speed running (19.8–25.2 km·h^−1^), sprinting (>25.2 km·h^−1^), high-speed activities (the distance covered in high-speed running and sprinting), the number of sprints (>25.2 km·h^−1^), accelerations (>3 m/s^2^), and decelerations (<−3 m/s^2^) as previously used (22, 23).

### 2.4. Statistical analyses

A statistical package (The jamovi project. (2022). jamovi. (Version 2.3) [Computer Software] https://www.jamovi.org) was used for the statistical analysis. A descriptive statistic with mean and standard deviation (SD) was performed for all variables. A linear mixed model was used to examine the differences in external load variables between T30 and T45, accounting for individual repeated measures. A general linear model was used to analyse the differences between the actions and mean stopped time between games T30 and T45. One sample proportion test was used to compare the offensive technical-tactical variables between the two conditions. Cohen's d effect sizes and 95% confidence intervals (CI) were performed, and interpreted as follows: <0.2 = trivial; 0.20 = small; 0.50 = moderate; and 0.80 = large (24). A significance level was set at *p* < 0.05.

## 3. Results

T30 presented a significantly higher total (*p* = <.001; ES 95% [CI] = 0.76 [0.48–1.03]), effective [*p *= <.001; ES 95% CI = 0.62 (0.34–0.89)], and stopped [*p* = 0.003; ES = 95% CI: 0.38 (0.12–0.65)] time compared to T45.

Table 2 depicts the absolute stopped time during the games. Table 3 presents the comparison between T30 and T45 for mean stopped time. In T30, there was significantly more time spent on kick-offs [*p* = 0.22; ES 95% CI = 0.88 (0.08–1.67)] compared to T45.

**Table 2:** Absolute data of match events related with stopped time during the games.

Actions	All games	Game 1		Game 2		Game 3		Game 4		Game 5		Game 6	
	(min:sec)	(min:sec)		(min:sec)		(min:sec)		(min:sec)		(min:sec)		(min:sec)	
	Mean	*n*	Total time	*n*	Total time	*n*	Total time	*n*	Total time	*n*	Total time	*n*	Total time
	(min-max)												
Medical assistance	01:13 (00:04–02:50)	6	08:54	1	00:49	2	03:31	5	05:58	5	05:27	2	01:09
Goal-kick	00:16 (00:02–00:48)	15	05:29	21	04:43	13	02:57	20	06:24	16	05:11	18	03:40
Corner	00:26 (00:09–00:42)	10	04:29	6	02:50	8	03:37	10	04:49	5	01:50	10	04:17
Throw-in	00:12 (00:00–01:50)	58	10:41	59	15:39	41	07:53	45	07:19	45	09:51	39	09:02
Kick-off	00:41 (00:23–01:21)	8	04:32	5	04:43	4	02:28	6	04:07	4	02:23	3	02:24
Free-kick	00:22 (00:02–01:15)	54	21:36	25	07:37	28	08:52	34	10:57	33	14:35	27	10:33
Substitutions	00:43 (00:03–1:59)	2	02:27	–	–	–	–	–	–	3	01:26	1	00:29

**Table 3:** Comparison of the mean stopped time between T30 and T45.

Actions	T30		T45		*p*-value	ES (95% CI)
	*n*	Mean	*n*	Mean		
		(min-max)		(min-max)		
Medical assistance	9	01:32 (00:46–02:50)	12	00:59 (00:04–02:39)	0.211	0.57 (−0.35–1.46)
Goal-kick	53	00:17 (00:02–00:37)	50	00:15 (00:02–00:48)	0.562	0.11 (−0.27–0.50)
Corner	22	00:27 (00:09–00:42)	27	00:26 (00:15–00:38)	0.643	0.13 (−0.43–0.69)
Throw-in	157	00:13 (00:02–01:50)	130	00:12 (00:00–00:30)	0.514	0.07 (−0.15–0.31)
Kick-off	14	00:46 (00:32–01:21)	16	00:37 (00:23–00:53)	0.022*	0.88 (0.08–1.67)
Free-kick	111	00:21 (00:02–01:15)	90	00:23 (00:02–01:02)	0.406	−0.11 (−0.39–0.16)
Substitutions	3	01:05 (00:28–01:59)	3	00:22 (00:03–00:35)	0.221	1.18 (−0.82–3.03)

General linear mixed model was used to compare the differences between T30 and T45.

* *p*-value was set <0.05.

Table 4 describes a comparison between T30 and T45 for offensive technical-tactical variables. During T30 players had significantly higher total possession (*p* = 0.002) and 1/3 possession (*p* = 0.018) compared to T45.

**Table 4:** Comparison of offensive technical-tactical variables between T30 and T45.

Tactics	T30		T45		*p*-value
	*n*	%	*n*	%	
Total possession	703	54,4	590	45,6	0.002*
1/3 Possession	276	55,3	223	44,7	0.018*
Within area	65	49,6	66	50,4	0.930
Shot within area	46	50,5	45	49,5	0.917
Shot out of area	43	47,3	48	52,7	0.600

Proportion test was used to analyze the game's technical-tactical characteristics between T30 and T45.

* *p-*value was set <0.05.

Regarding external load metrics, players covered a significantly higher total distance [*p* = <.001; ES 95% CI = 0.71 (0.44–0.99)], moderate-speed running [*p* = 0.002; ES 95% CI = 0.26 (0.00–0.52)], high-speed running [*p* = <.001; ES 95% CI = 0.33 (0.07–0.60)], and performed more high-speed activities [*p* = 0.003; ES 95% CI = 0.24 (−0.01–0.50)], accelerations [*p* = <.001; ES 95% CI = 0.32 (0.05–0.58)] and decelerations [*p* = 0.005; ES 95% CI = 0.26 (0.05–0.52)] during T30 compared to T45 (Table 5).

**Table 5:** Comparison of the total, effective, and stopped time and external load metrics between T30 and T45.

Variables	T30	T45	*p*-value	ES (95% CI)
Time				
Total time (min)	49:30 ± 08:00	45:00 ± 00:00	<.001*	0.76 (0.48–1.03)
Effective time (min)	28:70 ± 03:00	26:80 ± 03:10	<.001*	0.62 (0.34–0.89)
Effective time (%)	59:40 ± 10:20	59:50 ± 07:00	0.911	−0.01 (−0.27–0.24)
Stopped time (min)	20:60 ± 08:10	18:20 ± 03:10	0.003*	0.38 (0.12–0.65)
External load				
Total distance (m)	4,899 ± 610	4,481 ± 550	<.001*	0.71 (0.44–0.99)
Moderate-speed running (14.4–19.8 km·h^−1^) (m)	607 ± 183	557 ± 195	0.002*	0.26 (0.00–0.52)
High-speed running (19.8–25.2 km·h^−1^) (m)	202 ± 96.3	170 ± 89.1	<.001*	0.33 (0.07–0.60)
Sprinting (>25.2 km·h^−1^) (m)	45.9 ± 43.3	41.8 ± 42.1	0.619	0.09 (−0.16–0.35)
High-speed activities (19.8 km·h^−1^)^^^ (m)	284 ± 159	245 ± 154	0.003*	0.24 (−0.01–0.50)
Sprints (*n*)	2.3 ± 2.2	2.1 ± 2.1	0.345	0.10 (−0.15–0.37)
Accelerations (>3 m·s^2^) (*n*)	27.0 ± 11.0	24.0 ± 9.0	<.001*	0.32 (0.05–0.58)
Decelerations (>3 m·s^2^) (*n*)	31.0 ± 11.0	28.0 ± 11.0	0.005*	0.26 (0.00–0.52)

Linear mixed model was used to compare the differences between T30 and T45.

CI, confidence interval; ES, effect size; ET, effective time; m, meters; n, numbers; T30, 30 min half-game with stopped time; T45, 45 min half-game.

* *p*-value was set <0.05.

^ High-speed activities includes high-speed running and sprinting.

## 4. Discussion

This experimental study aimed to explore the effects of effective playing time in several technical-tactical and physical parameters in football match. The main findings were that T30 presented higher total, effective, and stopped time when compared to T45. A total ball possession and 1/3 possession higher percentage in T30 compared to T45. Lastly, T30 presented higher external load metrics (i.e., total distance, moderate- and high-speed running, high-speed activities, accelerations, decelerations) when compared to T45. Therefore, the results of higher effective playing time may affect technical-tactical and physical parameters during football games.

Considering time related variables, T30 presented significant higher total, effective, and stopped time compared to T45. However, no significant differences were found when effective playing time was calculated as a percentage of the total time. To the author's knowledge this is the first experimental study that compares two different time protocols (T45 vs. T30) and time related variables. Furthermore, Errekagorri et al. (17) demonstrated that the total playing time increased slightly when compared to three situations (without the intervention of VAR = 95.1 min, one intervention of VAR = 96.0 min, and 2–3 interventions of VAR = 99.1 min), however, effective playing time decreased significantly with intervention of the VAR (51.5 min) when compared without VAR (52.5 min). Therefore, more research about the influence of player's behavior about time consuming during official matches with and without stopping the chronometer when the ball was out-of-play is warranted.

In recent years, it has been considered these technical actions as key determinants of performance (16, 25, 26). Bush et al. (27) identified limited influence from match-related contextual variables (i.e., game location, final result, and opponents' level) on technical parameters (i.e., number of passes and shots on goal); however, shots, shots on target, and assists were noted has technical actions that displayed high match-to-match variation, which indicates that these variables were sensitive measures. Differently, Liu et al. (28) suggest that match-to-match variation of players' technical performances is affected by different match contexts. Thus, caution should be taken when using these variables to assess and interpret players' match performance, for consideration, long-term analysis seems to lead to a decrease in the impact of match-related variables on technical parameters (29). In the current study total ball possession and 1/3 ball possession were significantly higher during T30 when compared to T45. Previous studies considered a greater possibility of finishing successfully the attack when the ball carrier is closer to the opponent's goal (30, 31). However, higher ball possession did not significantly affect the number and the proportion of shots within area and out of area, during T30 compared to T45. Considering technical-tactical parameters, a recent study (17) presented the total scored goals increased as the number of VAR intervention increased (without VAR intervention = 1.2 goals; one VAR intervention = 1.5 goals; 2–3 VAR interventions = 1.7 goals). Moreover, regarding offensive variables, it has been indicated that teams’ performance mainly results from the ability to manage the game, keep ball possession, and create shots opportunities (32). Currently, studies on the success of football teams showed that the effectiveness and quality of the shots rather than the number and quantity determine the final score of the match (25, 26, 28, 33–35).

Considering external match load metrics, players covered more total distance, moderate- and high-speed running, and performed more high-speed activities, accelerations and decelerations during T30 compared to T45. These results are in line with previous studies in elite and youth soccer players (12, 18–20). Specifically, effective playing time influenced physical match performance, with total distance and accelerations being the most affected (*r* = 0.48–0.61), and high-intensity and sprinting distance, and maximum velocity the least affected parameters (*r* = −0.17–0.03) (12). Furthermore, players covered on average 10% more total distance and performed 13% more accelerations, while sprinting 7–10% less in matches with long (>65 min) compared to short (<50 min) effective playing times (12). These results could be explained by the higher total and effective playing time. The results reported by Peñas et al. (34), are in line with the current study, that higher physical demands in the first half might be related with higher effective playing time when compared to the second half of the match (27.4 ± 2.2 min vs. 26.9 ± 2.4 min). Moreover, a recent study (13) found that effective playing time should be considered when interpreting the match running performance on professional soccer players, as non-significant differences on high-speed running and sprinting distances were found between the 1st and 2nd halves in professional soccer players when the effective playing time was considered. Lastly, another study (36) showed that total distance and relative total distance significantly decreased in seasons with VAR compared to seasons without VAR in the top Spanish professional football league. Consequently, higher effective playing time may increase the physical match demands, but more research is warranted on this topic.

Whilst this is the first experimental study to explore different time methods (T30 and T45) on effective playing time in football games, the experiment is not without limitations. Contextual factors such as the phase of the season (i.e., beginning, middle, or end), relative importance of the game, match location (i.e., home or away), match score, classification and the level of the opponent team, as well as players' position, were not considered. Another limitation is the number of games (*n* = 6) performed. In this study, the players, staff, and referees were unaware of the type of the game (T30 and T45) and the time regulation, and only two types of games were designed. More research about the effects of different time regulations on technical, tactical, physical, and psychological parameters is needed. Also, future studies can split the data into 15 min periods and consider the score influence.

In summary, T30 presented a significantly higher total time, effective playing time, and stopped time compared to T45. Players had significantly higher total ball possession and 1/3 ball possession, and covered a significantly higher total distance, moderate-speed running, high-speed running, and performed more high-speed activities, accelerations and decelerations in T30 compared to T45. Furthermore, these findings suggest that the effective playing time may have meaningful effects on technical-tactical and physical parameters during football games. Therefore, international organizations should have on mind that implementing a stopped clock during football matches, this regulation may change the teams and players' technical-tactical behavior, and consequently the match demands.

## Data Availability

The raw data supporting the conclusions of this article will be made available by the authors, without undue reservation.
